# Inactivated poliovirus type 2 vaccine delivered to rat skin via high density microprojection array elicits potent neutralising antibody responses

**DOI:** 10.1038/srep22094

**Published:** 2016-02-25

**Authors:** David A. Muller, Frances E. Pearson, Germain J.P. Fernando, Christiana Agyei-Yeboah, Nick S. Owens, Simon R. Corrie, Michael L. Crichton, Jonathan C.J. Wei, William C. Weldon, M. Steven Oberste, Paul R. Young, Mark A. F. Kendall

**Affiliations:** 1 Delivery of Drugs and Genes Group (D2G2), Australian Institute for Bioengineering and Nanotechnology, The University of Queensland, Brisbane, Queensland, Australia; 2 Australian Infectious Diseases Research Centre, The University of Queensland, Brisbane, Queensland, Australia; 3 Division of Viral Diseases, National Center for Immunization and Respiratory Diseases, Centers for Disease Control and Prevention, Atlanta, Georgia, USA; 4 School of Chemical and Molecular Biosciences, The University of Queensland, Brisbane, Queensland, Australia

## Abstract

Polio eradication is progressing rapidly, and the live attenuated Sabin strains in the oral poliovirus vaccine (OPV) are being removed sequentially, starting with type 2 in April 2016. For risk mitigation, countries are introducing inactivated poliovirus vaccine (IPV) into routine vaccination programs. After April 2016, monovalent type 2 OPV will be available for type 2 outbreak control. Because the current IPV is not suitable for house-to-house vaccination campaigns (the intramuscular injections require health professionals), we developed a high-density microprojection array, the Nanopatch, delivered monovalent type 2 IPV (IPV2) vaccine to the skin. To assess the immunogenicity of the Nanopatch, we performed a dose-matched study in rats, comparing the immunogenicity of IPV2 delivered by intramuscular injection or Nanopatch immunisation. A single dose of 0.2 D-antigen units of IPV2 elicited protective levels of poliovirus antibodies in 100% of animals. However, animals receiving IPV2 by IM required at least 3 immunisations to reach the same neutralising antibody titres. This level of dose reduction (1/40th of a full dose) is unprecedented for poliovirus vaccine delivery. The ease of administration coupled with the dose reduction observed in this study points to the Nanopatch as a potential tool for facilitating inexpensive IPV for mass vaccination campaigns.

In 1988, when the World Health Assembly resolved to eradicate poliomyelitis globally, wild poliovirus was endemic in over 125 countries, causing an estimated 350,000 cases of poliomyelitis each year^1^. Spearheaded by Global Polio Eradication Initiative (GPEI), the number of poliomyelitis cases was reduced by over 99% with polio now endemic in only two countries, Afghanistan and Pakistan^2^. In 1999, wild poliovirus type 2 was eradicated and currently there has not been a single case of wild type 3 poliovirus since 2012. The success of this program has largely been due to volunteer health workers delivering the oral poliovirus vaccine (OPV).

The GPEI has almost exclusively relied on the use of the attenuated poliovirus vaccines developed by Albert Sabin^3^. This vaccine is easy to administer, only requiring two drops of vaccine delivered orally^3^. OPV is an effective vaccine producing long lasting systemic and mucosal immunity, with 95% of recipients protected after three doses in industrialised countries, but a lower proportion in developing tropical countries. However, despite the global success of this vaccine there are also disadvantages. For example, in rare cases the attenuated virus itself can cause paralysis^4^,^5^. In addition, as it is an attenuated live virus, like its wild-type counterpart it replicates within the gut. After several rounds of replication, accumulation of mutations can restore the neurovirulent virus phenotype. As the virus is excreted in faeces, subsequent contact by naïve individuals can cause infection. These infections can lead to outbreaks of circulating vaccine-derived polioviruses (cVDPV). Since 2006, more than 97% of all cVDPV cases have been type 2 poliovirus. To eliminate the threat of cVDPV2, in countries where it is still used, trivalent OPV is to be withdrawn and replaced by vaccination with bivalent OPV (types 1 and 3) along with at least one dose of “killed” or inactivated polio vaccine (IPV)^4^,^6^. Production costs for IPV have been estimated to be at least five times as much per dose as OPV primarily because of the additional manufacturing processes required for virus inactivation and the need for trained professional healthcare workers to deliver the vaccine intramuscularly (IM). At some point after polio has been eradicated and circulation of wild-type polioviruses has ceased, all OPVs will be withdrawn and IPV will be the only vaccine for poliomyelitis prevention. However, this poses a challenge for mass vaccination campaigns (to control possible outbreaks of disease) since intramuscular IPV injections the need to be administered by trained health professionals. Therefore, the mass vaccination campaigns with injectable vaccines are usually conducted in fixed sites (i.e. usually, health centres). In addition, as more countries gradually become self-sufficient, the cost per dose becomes more important. To help reduce the cost of vaccination, many approaches are under consideration to reduce antigen and doses required – including using adjuvants, and unlocking the dose sparing potential of the skin using various intradermal injectors and skin patches^7^. Here we examine the delivery of IPV via a novel skin patch, called the “Nanopatch” as a viable alternative.

The Nanopatch is a high-density microprojection array (e.g. 10,000 cm^−2^, 230 μm in length; for the prototype used here on rats) made from silicon to deliver dry-coated vaccine into the skin. When vaccine-coated Nanopatches are applied to the skin dynamically (e.g. using a spring-loaded applicator)^8^, the Nanopatch has reproducibly targeted the vaccine to thousands of antigen-presenting cells in both the viable epidermal and dermal layers of the skin^8^,^9^,^10^. The combination of targeted vaccine together with inflammation resulting from localized cell death generated by the dynamic application of projections into the skin leads to improved immune responses over standard needle-based intradermal delivery (e.g. the Mantoux method)^11^.

In previous studies using the mouse model, we have shown that Nanopatch delivery to these layers of the skin results in enhanced immunogenicity. As one example, for a seasonal influenza vaccine, Nanopatch (NP) delivery resulted in 1:100 dose-sparing when compared to conventional IM immunisation^9^. More broadly, this approach has been successful for vaccines of differing types, including the human papilloma virus-like particle vaccine^12^, split virus (influenza)^9^,^13^, DNA plasmid (West Nile Virus)^10^ and live viral vectors (malaria)^14^. Additionally, co-delivery of antigen with adjuvant by Nanopatch has also achieved synergistically improved immune responses^13^,^15^.

In this study, we apply the Nanopatch to deliver monovalent IPV2 – compared with standard Intramuscular (IM) injection – using the Wistar rat model for poliovirus vaccine immunogenicity. We hypothesised that Nanopatch delivery would result in enhanced anti-poliovirus neutralising titres over IM injection.

## Results

### IPV2 formulation and Nanopatch coating

**Figure 1:**
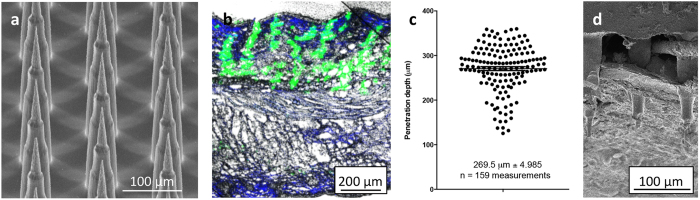
Nanopatch penetration analysis (**a**) SEM image of an uncoated Nanopatch; (**b**) fluorescent microscopy image of cryopreserved skin showing fluorescent microspheres deposited into the ear skin by the Nanopatch; (**c**) quantitative measurement of microsphere depth in skin (n = 159 measurements); (**d**) Representative Cryo SEM micrograph of Nanopatch projections in place within the viable epidermal and dermal layers of ear skin.

We began with establishing the coating formulation process for IPV2, to achieve a consistent coating across the Nanopatch surface and to maintain vaccine conformational stability throughout the coating/drying process. A limited screen of protectant sugars found the combination of 2% trehalose with 1% sucrose added to our standard 1% methylcellulose coating formulation resulted in a consistent coating. Silicon Nanopatches (4 × 4 mm), Fig. 1a, with 230 μm microprojections were dry-coated with the resultant IPV formulation and applied to the rat ear for 2 minutes. Following application, Nanopatches were removed and examined by SEM analysis, confirming the coating from the microprojections had been dissolved / removed from the surface of the microprojection.

### Penetration depth analysis

To confirm the vaccine was delivered to the epidermal and dermal layers of the skin, we performed a microprojection penetration study. Fluorescent microspheres were Nanopatch delivered and are visible in a representative histological section of rat ear skin showing green fluorescent micro-tracks spanning the length of the epidermis and dermis layers of the skin from the deposited microspheres (Fig. 1b). Quantitation of microsphere delivery showed that the microprojections penetrated on average, 269 μm ± 5 standard error of the mean into the rat ear (Fig. 1c). As the penetration depth is greater than the length of the 230 μm projections, it is likely that there is some compression of the ear skin during Nanopatch application (and also possibly some diffusion of the microspheres), resulting in deeper microsphere deposition similar to that observed previously^8^. Microprojection penetration was also investigated by CryoSEM analysis (Fig. 1d). The cross sectional view of a Nanopatch left in the ear confirms the microsphere analysis showing the microprojection spanning the full length of the epidermis and dermis.

### IPV2 immunogenicity study

**Figure 2:**
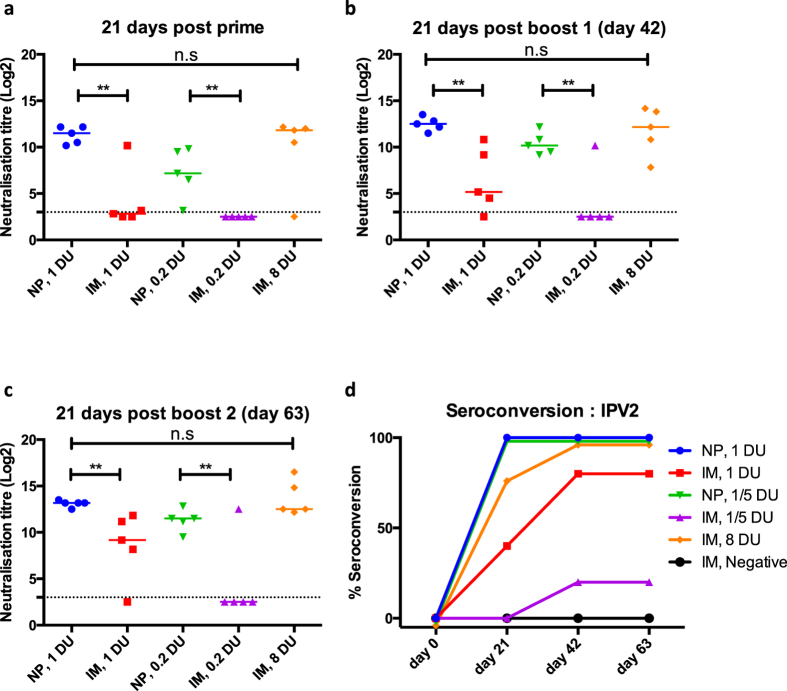
Neutralising antibody responses following IPV2 immunisation; (**a**) 21 days post priming; (**b**) 21 days post boost 1; (**c**) 21 days post boost 2; and (**d**) Seropositivity of each group to IPV2 immunisation over time, (seropositivity being defined as a neutralisation titre ≥3.0 log_2_ (dotted line)). Each symbol represents a single animal, with the line indicating the median titre. **indicates a statistically significant (*p* = <0.01) difference between dose matched Nanopatch and IM group as assessed by one-way ANOVA (alpha level 0.05) with a Tukey post test.

Following confirmation of appropriate penetration and vaccine delivery by the Nanopatch, we then examined the immunogenicity (antibody response) of the delivered IPV2. To investigate the dose sparing potential that has previously been observed in other studies with the Nanopatch, we performed a dose-matched comparative study. Wistar rats were immunised with three doses of 1 or 0.2 D-antigen units (DU) of IPV2 administered by Nanopatch or IM injection; 21 days apart. An additional group receiving three full human doses of 8 D-antigen units IM was included as the positive control. Prior to each dose, blood samples were collected; the sera of all rats were negative for neutralising antibodies against poliovirus type 2 at commencement of the study (Figure s1). Following a single dose of IPV2 (Fig. 2a), 100% of rats receiving 1 or 0.2 D-antigen units via the Nanopatch vaccine had positive poliovirus 2 neutralising antibody titres (median log_2_ titres = 11.5 or 7.17 respectively). In contrast, equivalent doses delivered by IM elicited protective levels of antibody in only two mice for the 1 D-antigen unit group (with significantly lower medians) and no seroconversion after a single 0.2 D-antigen units IM dose. Twenty per cent of animals receiving 0.2 D-antigen units by IM seroconverted following a booster immunisation at day 21 (Fig. 2b). Following the first immunisation, neutralising antibody titres continued to increase upon further immunisations for all groups (Fig. 2b,c), for IM groups receiving 8 D-antigen units (full human dose) showing 100% seroconversion (Fig. 2d). In contrast, animals immunised via the Nanopatch had significantly higher neutralising antibody titres after one or two doses of vaccine as determined by one-way ANOVA (alpha level 0.05) with a Tukey post-test (p = < 0.01). Of particular interest is that one D-antigen unit delivered by the Nanopatch elicited neutralising antibody titres that were not significantly different to those induced by 8 D-antigen units delivered by IM injection demonstrating at least 8-fold dose reduction by the Nanopatch. Using as little as 0.2 D-antigen units (1/40^th^ of a human dose) of IPV2 we were able to elicit protective levels (titre ≥ 3 log_2_) of neutralising antibodies from a single dose in 100% of animals (n = 5).

## Discussion

This is the first study demonstrating the dose sparing potential of the Nanopatch – or any other micropatch technology – with IPV. Here, we have shown that the dynamic application of the Nanopatch (i.e. using a spring loaded applicator) produces significant dose reduction in rats, with 1 or 0.2 D-antigen units of IPV2 eliciting 100% seroconversion after a single immunisation with significantly higher median neutralising antibody titres than those of the IM counterparts (fig. 2a–d). These results also show that the design changes made to the Nanopatch in moving from the mouse to the rat (an animal model approximately an order of magnitude heavier and with significantly thicker skin strata) result in a potent immune response from the skin, while still being a practical, simple device.

As poliovirus nears eradication, WHO has advised member countries to withdraw trivalent OPV and introduce immunisation with bivalent OPV (types 1 and 3)^16^, plus at least one dose of IPV to provide type 2 immunity, to avoid the risks of cVDPV (mostly associated with type 2)^17^,^18^. With the introduction of the more expensive IPV (approximately US$1/dose for GAVI countries^19^, compared with US$0.12–0.20 per dose for OPV^20^) the cost burden of mass and routine vaccination campaigns will increase^7^. As a result, WHO has investigated cost-saving measures to reduce antigen dose. One way to achieve this is with the use of adjuvants, such as dmLT^21^ or aluminium hydroxide^22^. Alternatively, approaches such as intradermal injection, microinjectors and microneedle patch technologies, are being explored to target the IPV vaccine to the skin which has been shown to be abundant in antigen presenting cells^7^. A number of studies investigating the use of ID vaccination have been conducted in humans and rat models, demonstrating that 1/5^th^ the dose of IPV can produce similar antibody responses or levels of protection (depending on the setting and immunisation regimen) as a full dose delivered by IM injection^23^,^24^,^25^,^26^,^27^,^28^,^29^,^30^,^31^. Despite the dose reduction found in these studies, IM injection of full-dose IPV continues as the preferred method of vaccination, due to its simplicity and reproducibility^7^. To improve the reproducibility of ID vaccination (e.g. the classic Mantoux method), a number of approaches have been evaluated. Microneedle delivery of liquid vaccine (e.g. the Nanopass device) has shown promise in preclinical and clinical trials^30^,^32^. Liquid-jet injectors (e.g. the Biojector 2000) also have the advantage of being needle-free, and have been shown to elicit >90% seroconversion in infants receiving two fractional doses of IPV in Cuban clinical trials^33^.

Recently, there have been several studies investigating the use of dissolving and solid microneedle technologies (arrays or single bioneedles), which have been shown to produce poliovirus-neutralising antibodies in Wistar rat and Rhesus macaque models. As one example, using a low density 1 cm^2^ microneedle array (600 μm in length), Edens *et al.* delivered a full human dose of IPV to Rhesus macaques, inducing similar responses to that of IM injection for types 1 and 2 polio^34^. Although responses to IPV3-patched animals was inferior to IM injection, the authors commented that this was likely due in part to incorrect D-antigen ELISA antibody selection. Other studies and our own unpublished work have noted the challenges associated with producing a stable formulation for IPV3^35^. Separately, using a single bioneedle, Kraan *et al.* elicited antibody responses to all three types of poliovirus, using a fractional dose (2.7–0.6–2.1 D-antigen units/dose) cast in a single implantable bioneedle–producing in Wistar rats at best similar neutralising antibody levels to the equivalent IM dose^35^. While both of these studies highlight the potential of solid formulated microneedles, neither demonstrated a dose reduction effect^34^,^35^ as a result of targeting the skin’s immune system.

Future studies will focus on developing our current Nanopatch to a delivery device for widespread vaccination with distinct advantages over existing needle-based approaches. Effectively translating the results presented in this study (i.e. significant dose-sparing) to humans could dramatically reduce the cost of, and stretch the constrained supply of IPV^36^, which is currently a major barrier for replacing OPV, and its use would potentially allow house-to-house administration of IPV by volunteers in mass immunization campaigns.

## Methods

### Inactivated polio vaccine formulation

Bulk concentrated inactivated poliovirus vaccine type 2 (763 D-antigens units/mL) was kindly provided by Bilthoven Biologicals, Bilthoven, the Netherlands.

### Nanopatch fabrication and coating

Solid Nanopatches were fabricated as described by Jenkins *et al.* (2012) at the Melbourne Centre for Nanofabrication^37^. The concentrated IPV2 was mixed with 1% methylcellulose, 2% trehalose and 1% sucrose (with some similarities in protocol as previously reported)^14^. Microprojections (10,000 cm^−2^) 230 μm in length were coated under a nitrogen jet stream as described by Chen, *et al.*^38^,^39^.

### Wistar rats

Specific pathogen-free female Wistar rats (6–8 weeks) were purchased from the Animal Resource Centre, Perth, and housed in the AIBN animal house facility. All methods performed in this study were carried out in accordance with National Health & Medical Research Council guidelines and approved by The University of Queensland Animal Ethics Committee.

### Nanopatch penetration analysis

Penetration depth analysis was performed as previously described by Muller, *et al.*^40^. Briefly, Nanopatches were coated with 0.4 μL of 200 nm FluroSpheres ® (Invitrogen, Cat# F8811) and applied as described above. Immediately following Nanopatch application, rats were euthanised and ears were collected and frozen in OCT medium. Cryosections of 20 μm thickness were analysed by two-photon microscopy and depth measurements were collected.

### CryoSEM analysis of Nanopatch skin sections

Visualisation of skin with intact projections was performed *in-situ* using CryoSEM with a Philips XL30 SEM (Philips, Netherlands) with an Oxford CT-1500 attachment as described in Crichton, *et al.*^8^. Briefly, patches were applied to rat skin and then mounted with the patch in-place vertically in a holder surrounded by Tissue-Tek OCT compound (Sakura, Netherlands). This assembly was frozen in liquid nitrogen and then removed under vacuum and entered into the SEM preparation chamber where both the skin and patch were fractured using a scalpel. Finally, the sample was sputter coated with a thin (nanometers) layer of gold before imaging at 5 kV in the SEM.

### D-antigen ELISA

A subtractive D-antigen ELISA was performed to determine the dose delivered by Nanopatch. The dose delivered into the ear was determined by eluting the coated vaccine from patches post application compared to coated, unapplied Nanopatches. The overall loss of D-antigen units compared to the unpatched samples represents the amount of delivered vaccine. The ELISA was performed as described by Edens *et al.* (2015)^34^,^41^. Briefly, Nunc Maxisorp plates were coated with 50 μl of anti-polio 2 antibody (Thermo Fisher Scientific Cat PIEHYB294-06-02), diluted 1:1,000 in carbonate-bicarbonate buffer pH 9.6 overnight at 4 °C. Plates were blocked for 1 hour at 37 °C in a humidified chamber in PBS-gelatine (0.5% gelatin (LabChem Cat #9000-70-8), 0.25% Tween-20 (Sigma Cat# P2287-500ML, 100 mL 0.01 M PBS). Following blocking, plates were washed three times in PBS 0.05% Tween-20. Eluted samples and international IPV standards (Cat# 12/104, NIBSC) were serially diluted in blocking buffer incubated for 1 hour at 37 °C in a humidified chamber. Plates were washed again and IPV2 detected by anti-polio HRP diluted 1:1,000 in blocking buffer and incubated for 1 hour at 37 °C in a humidified chamber. Plates were then washed a further four times before adding 50 μl of TMB and incubating for 6 minutes at room temperature protected from light. The reaction was stopped by the addition of 50 μl 1 M phosphoric acid and absorbance was read spectrophotometrically at 450 nm. D-antigen units were then calculated relative to a standard curve.

### Rat immunisation study

Immunisation regime was based on similar studies performed investigating fractional IPV responses^21^,^32^,^35^.Under ketamine hydrochloride (Ceva) and xylazine hydrochloride (Troy Laboratories) sedation, rats (n = 5) were immunised with 1 or 0.2 D-antigen units of IPV 2 by Nanopatch or IM injection, with an additional positive-control group receiving a full human dose of 8 D-antigen units IM. Two Nanopatches, each containing half the dose to be delivered were applied to each ventral ear pinnae using a proprietary applicator at a velocity 3.1 ms^−1^ and kept in place for 2 minutes. IM injections were administered into the hind leg by a 29G needle. Rats received three doses by Nanopatch or IM injection, at 21-day intervals, with blood samples collected one day before vaccination and 21 days post final dose.

### Polio virus neutralisation assay

To evaluate the immune response elicited by vaccination, poliovirus neutralisation assays were performed at the CDC as previously published by WHO^41^,^42^,^43^. Briefly, all samples were diluted 1 in 4, mixed with 80–100 CCID_50_ of each poliovirus (Sabin 1, 2 or 3) and incubated for 3 hours at 35 °C, then added to HEp-2c cells (7,500 cells per well) in 96-well plates. Plates were incubated at 35 °C, 5% CO_2_ for 5 days at which point cells were stained with crystal violet and CPE measured by optical density at 570 nm. Neutralisation titres were reported as reciprocal 50% endpoint titres determined by the Spearman-Karber method^44^. Positive samples are defined as having ≥ 3.0 log_2_ titre with an assay background of 2.5 log_2_ titre.

### Statistical analysis

All statistical analysis was performed using Graphpad Prism version 6.0f. Multiple comparison analysis was performed using one-way ANOVA with the alpha level set at 0.05 and a Tukey post-test.

## Additional Information

**How to cite this article**: Muller, D. A. *et al.* Inactivated poliovirus type 2 vaccine delivered to rat skin via high density microprojection array elicits potent neutralising antibody responses.. *Sci. Rep.* **6**, 22094; doi: 10.1038/srep22094 (2016).

## Supplementary Material

Supplementary Information
