# Supplementary material for: Inactivated poliovirus type 2 vaccine delivered to rat skin via high density microprojection array elicits potent neutralising antibody responses
Source: Sci Rep. 2016 Feb 25;6:22094. doi: 10.1038/srep22094 (PMC4766532; doi:10.1038/srep22094)
Supplement: Supplementary Information [file srep22094-s1.pdf]

**Title: Inactivated poliovirus type 2 vaccine delivered to rat skin via high density microprojection array elicits potent neutralising antibody responses**

Authors names and affiliations:

David A. Muller[1,2], Frances E. Pearson[1], Germain J.P. Fernando[1,2], Christiana Agyei-Yeboah[1], Nick S. Owens[1], Simon R. Corrie[1,2], Michael L. Crichton[1], Jonathon C.J. Wei[1], William C. Weldon[3], M. Steven Oberste[3], Paul R. Young[2,4], Mark A. F. Kendall[1,2*].

[1]Delivery of Drugs and Genes Group ($D^2G^2$), Australian Institute for Bioengineering and Nanotechnology, The University of Queensland, Brisbane, Queensland, Australia.

[2]Australian Infectious Diseases Research Centre, The University of Queensland, Brisbane, Queensland, Australia.

[3] Division of Viral Diseases, National Center for Immunization and Respiratory Diseases, Centers for Disease Control and Prevention, Atlanta, Georgia, USA.

[4]School of Chemical and Molecular Biosciences, The University of Queensland, Brisbane, Queensland, Australia.

***Corresponding author:** Professor Mark Kendall

Address: The University of Queensland, Delivery of Drugs and Genes Group, Australian Institute for Bioengineering and Nanotechnology, Building 75, St Lucia, Queensland, 4072. Australia

Tel: +61 7 334 64203   Fax: +61 7 334 64197

Email: m.kendall@uq.edu.au

Supplementary data:

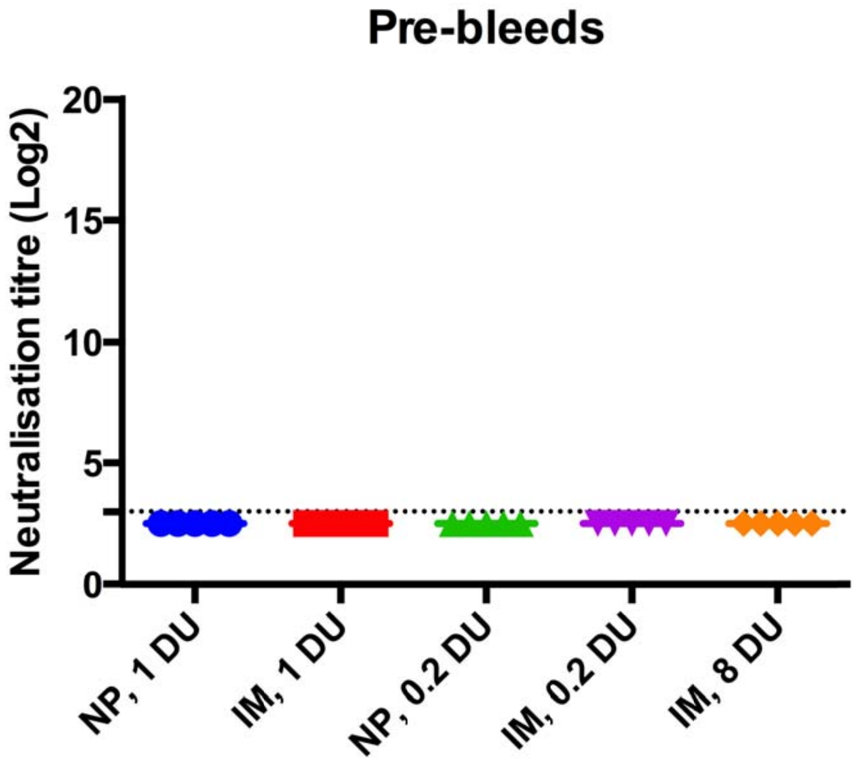


**Figure S1: Neutralising antibody titres prior to immunisation.** Seropositivity was defined as a titre ≥ 3.0 $log_2$ (the dotted line)
